# Crucial Role of Heme Oxygenase-1 on the Sensitivity of Cholangiocarcinoma Cells to Chemotherapeutic Agents

**DOI:** 10.1371/journal.pone.0034994

**Published:** 2012-04-13

**Authors:** Sarinya Kongpetch, Veerapol Kukongviriyapan, Auemduan Prawan, Laddawan Senggunprai, Upa Kukongviriyapan, Benjaporn Buranrat

**Affiliations:** 1 Department of Pharmacology, Faculty of Medicine, Khon Kaen University, Khon Kaen, Thailand; 2 Department of Physiology, Faculty of Medicine, Khon Kaen University, Khon Kaen, Thailand; 3 Liver Fluke and Cholangiocarcinoma Research Center, Khon Kaen University, Khon Kaen, Thailand; 4 Faculty of Pharmaceutical Sciences, University of Phayao, Phayao, Thailand; Oxford University, United Kingdom

## Abstract

Cancer cells acquire drug resistance via various mechanisms including enhanced cellular cytoprotective and antioxidant activities. Heme oxygenase-1 (HO-1) is a key enzyme exerting potent cytoprotection, cell proliferation and drug resistance. We aimed to investigate roles of HO-1 in human cholangiocarcinoma (CCA) cells for cytoprotection against chemotherapeutic agents. KKU-100 and KKU-M214 CCA cell lines with high and low HO-1 expression levels, respectively, were used to evaluate the sensitivity to chemotherapeutic agents, gemcitabine (Gem) and doxorubicin. Inhibition of HO-1 by zinc protoporphyrin IX (ZnPP) sensitized both cell types to the cytotoxicity of chemotherapeutic agents. HO-1 gene silencing by siRNA validated the cytoprotective effect of HO-1 on CCA cells against Gem. Induction of HO-1 protein expression by stannous chloride enhanced the cytoprotection and suppression of apoptosis caused by anticancer agents. The sensitizing effect of ZnPP was associated with increased ROS formation and loss of mitochondrial transmembrane potential, while Gem alone did not show any effects. A ROS scavenger, Tempol, abolished the sensitizing effect of ZnPP on Gem. Combination of ZnPP and Gem enhanced the release of cytochrome c and increased p21 levels. The results show that HO-1 played a critical role in cytoprotection in CCA cells against chemotherapeutic agents. Targeted inhibition of HO-1 may be a strategy to overcome drug resistance in chemotherapy of bile duct cancer.

## Introduction

Cholangiocarcinoma (CCA) is a malignant tumor of the bile duct, which originates from the bile duct epithelial cells (cholangiocytes). CCA is a devastating malignancy with poor prognosis. CCA is a rare type of cancer worldwide, however populations residing in the Southeast Asian region are at very high risk. The important risk factors are liver fluke infection and possible involvement from chronic infection with hepatitis B and C viruses [1], [2]. Early diagnosis and extensive surgery offers the only chance for prolonged life. Unfortunately, most patients are diagnosed at the advanced stage of disease and current biomarkers are of limited value [3], [4]. Chemotherapy is a remaining option. However, current chemotherapy has not been shown to substantially improve survival in patients with unresected CCA [3], [5]. Many chemotherapeutic drugs as well as targeted chemotherapeutic agents have been tested as single agents or in combinations. Nevertheless, drug resistance or drug inefficacy remain major obstacles in the treatment of CCA [6]. It is apparent that a new strategy of chemotherapeutic treatment is urgently needed in management of the unresectable CCA.

Heme oxygenase-1 (HO-1) is one of the powerful cytoprotective enzymes. HO-1 plays critical roles in physiological iron homeostasis, antioxidant defense, anti-inflammatory and anti-apoptotic effect [7]. It is induced by various stimuli such as hypoxia, UV-radiation, heavy metals, chemotherapeutic drugs and oxidative stress [8], [9]. HO-1 catalyzes the first and rate-limiting step in the degradation of heme to biliverdin, carbonmonoxide (CO) and ferrous iron. Biliverdin is further converted to bilirubin by biliverdin reductase. Biliverdin and bilirubin are the most potent endogenous reactive oxygen species (ROS) scavengers [7]. CO is also an efficient anti-inflammatory mediator in several models of inflammation and tissue injury [10], [11]. The increased expression of HO-1 has been observed in several cancers including brain tumor, melanoma, chronic myeloid leukemia, and lymphosarcoma [12], suggesting possible contribution of HO-1 to tumor progression through promotion of angiogenesis, metastases and pro-proliferation [13]. HO-1 expression may contribute to resistance to chemotherapeutic agents such as cisplatin, doxorubicin and gemcitabine in some human cancers [9], [14], [15]. Thus, some studies revealed that suppression of HO-1 activity or HO-1 knockdown by siRNA increased the chemosensitivity of AML cells, pancreatic and lung cancer cells [9], [14], [16], but was not effective in other cancer cells [17]. The inhibition of HO-1 by zinc protoporphyrin IX (ZnPP) induced apoptotic cell death and this may be associated with the increase in ROS production. Similarly, HO-1 gene silencing by specific siRNA also induced ROS generation [17]. However, the exact mechanism of the sensitizing effect to chemotherapeutic agents confered by suppression of HO-1 is largely unknown. Mitochondria may be a primary target of HO-1 inhibition, as ZnPP and triiodothyronine induced the opening of the mitochondrial permeability transition (MPT) pore leading to liver injury [18].

In the present study, we investigated whether HO-1 in CCA cells plays a critical role in cytoprotection against chemotherapeutic agents. The results show that inhibition of HO-1 induced the sensitization of CCA cells to gemcitabine (Gem) and doxorubicin (Dox). Inhibition of HO-1 could be a strategy to enhance the response of CCA to chemotherapeutic drugs.

## Materials and Methods

### Cell lines and cell cultures

The human cholangiocarcinoma (CCA) cell lines; KKU-100 and KKU-M214 used in this study were kindly provided by Dr. Banchob Sripa of Department of Pathology, Faculty of Medicine, Khon Kaen University. Both cell lines were cultured in complete media consisting of Ham's F12 media, supplemented with 10% fetal calf serum, 12.5 mM HEPES, pH 7.3, 100 U/ml penicillin G and 100 µg/ml gentamicin. The cells were subcultured every 3 days using 0.25% trypsin-EDTA and the medium was renewed after an overnight incubation. The cultured cells were changed to incubate in serum-free defined Ham's F12 medium immediately before further treatment.

### Cytotoxicity assay

Cytotoxicity was determined by fluorescent staining using acridine orange and ethidium bromide (AO/EB) as described previously [19]. KKU-100 (7,500 cells/well) and KKU-M214 (5,000 cells/well) cells were cultured in 96 well-plate and allowed to attach overnight. On the next day, the medium was removed and gemcitabine (Gemzar®, Eli Lilly, IN, USA: Gem) dissolved in phosphate-buffered saline (PBS: 137 mM NaCl, 2.7 mM KCl, 10 mM Na_2_HPO_4_, 2 mM KH_2_PO_4_, pH 7.4), doxorubicin HCl (Boryung Pharm, Seoul, South Korea: Dox) dissolved in DMSO (100 mM) and further diluted with PBS, 4-hydroxy-2,2,6,6-tetramethylpiperidine-1-oxyl (Tempol), dissolved in PBS, zinc protoporphyrin IX (ZnPP; HO-1 inhibitor), dissolved in DMSO (50 mM) and further diluted with PBS or stannous chloride (SnCl_2_; HO-1 inducer) dissolved in PBS, or combinations of them were added to the media culture to final concentrations as indicated in results section and incubated for a designated period of time. Then, cells were washed once with PBS and stained with AO/EB. The cells were examined using a Nikon Eclipse TS100 inverted microscope with excitation and long–pass emission filters of 480 and 535 nm, respectively. The fluorescent images were taken at predetermined areas with a Nikon Coolpix digital camera. The numbers of viable, apoptotic and necrotic cells which were stained with green fluorescence, bright orange fluorescence and green fluorescence with appearance of cell shrinkage, condensation and fragmentation of the nuclei, respectively, were enumerated. The cytotoxicity value was calculated as = (number of viable cells in treatment wells)/(number of viable cells in control cells)×100.

### HO-1 small interfering RNA transfection

The transfection of HO-1 siRNA was performed using siGENOME SMARTpool (M-006372-02-0005: Dharmacon, CO, USA) at a final concentration of 200 nM and lipofectamine 2000 (Invitrogen, CA, USA) according to the manufacturers' instructions. HO-1 expression was specifically suppressed by introduction of siRNA against human HMOX1 (Gene Id: 3162). - In brief, cells were grown in a 6-well plate to reach a confluence of 70%. For each well, 100 pmoles of HO-1 siRNA were mixed with 2 µL of lipofectamine 2000 and 500 µL of serum- and antibiotic-free Ham's F12 medium was added. Cells were exposed to the transfection mixture for 6 h. At the end of incubation period, 1.5 mL of antibiotic-free Ham's F12 complete medium was added and the cells were cultured for an additional 18 h. The siGENOME non-targeting siRNA (D-001210-02-05: Dharmacon), as a negative control was introduced to the cells using the same protocol. Total RNA and protein were extracted from cells 24 h after transfection using previously described methods [20], [21]. Efficiency of the transient transfection was determined by real-time polymerase chain reaction (PCR) using specific primers and Western blotting.

Cytotoxicity of Gem to HO-1 knock-down CCA cells was performed. KKU-100 cells (7,500 Cells/well) were grown in 96-well plate to reach the confluence of 70%. For each well, cells were transfected with 3 ρmoles of HO-1 siRNA reagent mixed with 0.06 µL of lipofectamine 2000 in serum- and antibiotic-free Ham's F12 medium. Cells were kept in the transfection mixture for 6 h. Then, 100 µL of antibiotic-free Ham's F12 complete medium was added and the cells were kept for an additional 18 hours. Then, cells were treated with varied concentrations of Gem for further 24 h and cytotoxicity assay was performed as described as above.

### Real-time polymerase chain reaction

KKU-100 and KKU-M214 cells were seeded at the density of 1.5×10^5^ cells/well in 6 well-plates and allowed to growth for 24 h. Total RNA was isolated using a previously described method [20]. Total RNA (1 µg) was then reverse transcribed to single-stranded cDNA by the ImProm-II™ reverse transcription system (Promega, WI, USA) at conditions of 42°C for 60 min. The reverse transcription products served as a template for real-time PCR. The primer sequences were as follows: HO-1: forward, 5′-CTG ACC CAT GAC ACC AAG GAC-3′ and HO-1 reverse: 5′-AAA GCC CTA CAG CAA CTG TCG-3′, β-actin: forward 5′-AGT GTA GCC CAG GAT GCC CTT-3′ and β-actin: reverse, 5′-GCC AAG GTC ATC CAT GAC AAC-3′. The PCR was performed in a final volume of 15 µL containing cDNA template, 5 µM of each HO-1 primer or 2.5 µM of each β-actin primer in SsoFast™ EvaGreen Supermix with low Rox (Bio-Rad, CA, USA). After an initial denaturation step at 95°C for 10 min, 40 cycles for HO-1 and β-actin were performed as follows: denaturating for 15 sec at 95°C, annealing for 30 sec at 55°C and extension for 45 sec at 72°C. To verify the purity of the products, a melting curve analysis was performed after each run. To quantify the relative expression of genes, the relative quantitation using standard curve method was used. The amount of HO-1 mRNA was expressed as a ratio to β-actin mRNA.

### Western blot analysis

Western blot analysis was used to determine the expression levels of HO-1, p21^Cip/WAF1^, cytochrome C, and β-actin. KKU-100 (7.5×10^5^) and KKU-M214 (6×10^5^) cells were cultured in 100 mm^3^ dishes and treated with drug or drug combinations. The cultured cells were washed with PBS, lysed with RIPA buffer [150 mM NaCl, 1% NP-40, 0.5% sodium deoxycholate, 0.1% SDS, 50 mM Tris–HCl (pH 7.4), 50 mM glycerophosphate, 20 mM NaF, 20 mM EGTA, 1 mM DTT, 1 mM Na_3_VO_4_ and protease inhibitor cocktail (M221: Amresco, OH, USA) at 4°C for 15 min and transferred into a microtube. After vigorous vortex mixing, the suspension was centrifuged at 12,000×g for 20 min and supernatant was collected and stored at −70°C until use. The protein samples were mixed with SDS loading buffer and subjected to separation by electrophoresis in 8–10% SDS-polyacrylamide gel. The bands were blotted onto a PVDF membrane. The membranes were blocked for 1 h at room temperature with 5% (w/v) skimmed milk powder in Tris buffered saline (TBS) containing 0.1% Tween-20. The PVDF membrane was incubated overnight at 4°C with primary antibodies of rabbit polyclonal anti-human HO-1 (1∶1000) (ADI-SPA-895: Enzo Life Sciences, Switzerland), rabbit monoclonal anti-human p21^Cip/WAF1^ (1∶500) (#2947: Cell Signaling Technology, MA, USA), mouse monoclonal anti-human cytochrome c (1∶1000) (sc-13560) and horseradish peroxidase (HRP)-goat polyclonal anti-human β-actin (1∶2500) (sc-1616 HRP) in TBS. After washing with TBS the blots were incubated for 1 h at room temperature with the HRP-conjugated secondary antibodies (anti-rabbit IgG-HRP sc-2004, anti-mouse IgG-HRP sc-2005, anti-goat IgG-HRP sc-2354). After removal of the secondary antibody and TBS buffer washes, the blots were incubated in ECL substrate solution (SuperSignal West Pico Chemiluminescent Substrate: Thermoscientific, IL, USA). The densities of the specific protein bands were visualized and captured by ImageQuant™ 400.

### Measurement of intracellular ROS accumulation

To monitor the intracellular accumulation of ROS, the lucigenin-enhanced chemiluminescence method was used for detecting superoxide anion according to the previously described method [20]. Briefly, KKU-100 cells were cultured in 35-mm dishes overnight. After treatment with compounds for 3 h, cultured cells were rinsed, incubated in PBS containing lucigenin and measured for luminescent signal in a 20/20 n Luminometer (Turner Biosystem, CA, USA).

### Measurement of mitochondrial transmembrane potential

To analyze the mitochondrial transmembrane potential (Δψ_m_), the lipophilic cationic fluorescent probe; JC-1 (Cayman Chemical, MI, USA) was used as previously described [21]. KKU-100 cells were seeded in a 96-well plate for an overnight before treatment with compounds for 6 or 24 h. Cultured cells in the plate were centrifuged at 1,500 rpm 25°C for 5 min and were loaded with JC-1 dye for 30 min at 37°C. Then, cultured cells were rinsed, incubated in JC-1 assay buffer and Δψ_m_ was analysed in a fluorescent microscope. JC-1 forms J-aggregates in cells with healthy mitochondria, which can be detected with fluorescent settings of excitation and emission wavelengths at 560 and 595 nm, respectively. Cells with depolarized mitochondria, JC-1 existed as J monomers can be detected with excitation and emission wavelengths at 485 and 535 nm, respectively. The change of the orange fluorescence in healthy cultured cells to green fluorescence is an indicative of depolarization of Δψ_m_.

### Statistical analysis

Data are expressed as mean ± SEM of three separated experiments. An analysis of variance was used to determine significant differences between each experimental group. The level of significance was set at *P*<0.05.

## Results

### Basal HO-1 expression and inducible expression by anticancer agents

**Figure 1:**
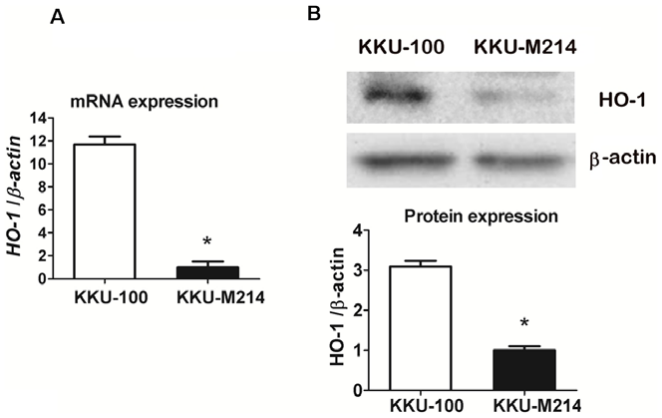
HO-1 mRNA and protein expressions in CCA cells. (**A**) The basal mRNA expression of HO-1 in CCA cells; KKU-100 and KKU-M214 cells. Total RNA of cells were collected and analysed by real-time RT-PCR. The bars represent relative expression of HO-1 normalized with β -actin. The expression of HO-1 in KKU-100 cells is much higher than that of KKU-M214 cells, p<0.05. (**B**) The basal protein expression of HO-1 in KKU-100 and KKU-M214 cells. Total cell lysates were prepared and subjected to Western blot analysis using β-actin as a loading control. Image samples of HO-1 and β -actin are shown in the top panel of the figure. HO-1 protein in KKU-100 cells is relatively higher than that of KKU-M214 cells. The bars represent mean±SEM, each from three separated experiments. *, *p*<0.05 vs KKU-100 group.

**Figure 2:**
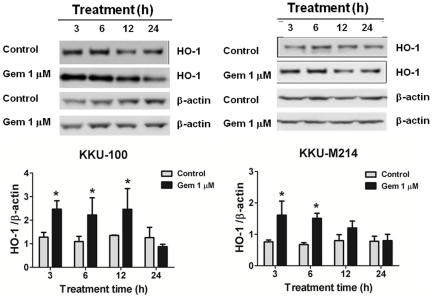
Time-course of HO-1 protein induction by Gem. (**A**) KKU-100 cells and (**B**) KKU-M214 cells were cultured for overnight and exposed to Gem (1 µM) for 3, 6, 12 and 24 h. The cultured cells exposed to vehicle only were performed as concurrent control groups. Total cell lysates were collected at the indicated time points and subjected to Western blot analysis using β-actin as a loading control. The relative expression of HO-1 was normalized with β actin. The bars show the HO-1 expression in Gem-treated group and concurrent control group. The bars represent mean±SEM, each from three separated experiments. *,*p*<0.05 vs concurrent controls.

The two cholangiocarcinoma cells, KKU-100 and KKU-M214, were used to determine the basal expression levels of HO-1. The mRNA and protein expressions of HO-1 were determined by real-time PCR and Western blot analysis. At basal condition, KKU-100 showed the higher HO-1 mRNA and protein levels than KKU-M214 cells (Fig. 1A–B). To examine whether anticancer agent could induce HO-1 in CCA cells, two lines of cells were incubated with 1 µM of Gem and the time-course of HO-1 protein expression was examined. Both KKU-100 and KKU-M214 cells treated with Gem showed a rapid increased HO-1 protein expression within 3 h when compared with concurrent controls and returned to control level by 24 h (Fig. 2).

### Effect of HO-1 inhibition on the sensitivity of CCA cells to anticancer agents

**Figure 3:**
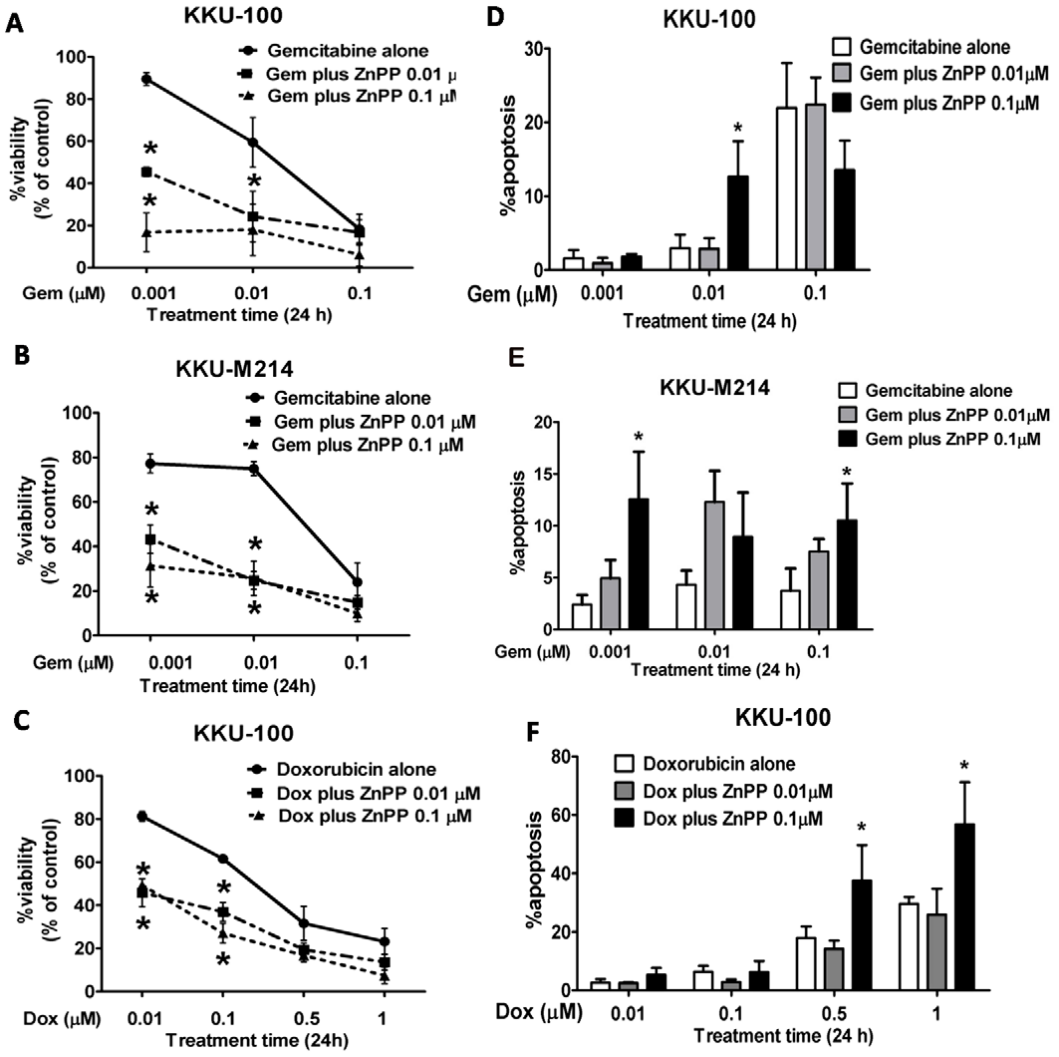
Inhibition of HO-1 activity enhances the cytotoxic effect of Gem. KKU-100 and KKU-M214 cells were treated with the varied concentrations (0.001, 0.01 and 0.1 µM) of Gem (A,B,D and E) and (0.01, 0.1, 0.5 and 1 µM) of Dox (C and F) with or without 0.01 and 0.1 µM of ZnPP (HO-1 inhibitor) for 24 h. KKU-100 and KKU-M214 cells were evaluated for cytotoxicity (A,B and C) and apoptosis (D,E and F) by fluorescent dye staining. The cytotoxicity of the drugs are in concentration-response manner, whereas in co-treatment of drug and ZnPP, the concentration-response curves are shifted downward, indicating enhancement of the response to drug combinations. The bars at the right panel show percent of apoptotic cell death when cells were treated with anticancer drugs in combination with various concentrations of ZnPP (0.001–0.1 µM). The bars represent mean±SEM, each from three separated experiments. *, *p*<0.05 vs drug alone (Gem and Dox).

The cytoprotective effects of HO-1 in CCA cells to anticancer agents were explored using high and low HO-1 expressing KKU-100 and KKU-M214 cells cultured with HO-1 inhibitor. Both cells were exposed to Gem (0.001–0.1 µM) in the presence of HO-1 inhibitor, ZnPP (0.01 and 0.1 µM) for 24 h. As shown in Fig. 3A and 3B, ZnPP rendered both CCA cells to be highly susceptible to cytotoxic effect of Gem, which can be seen as the downward shift of the dose-response curves of Gem in the presence of ZnPP. Similar downward shift of the dose-response curves in the presence of ZnPP was observed in KKU-100 cells to another chemotherapeutic agent, Dox (Fig. 3C). The presence of ZnPP augmented significantly Gem- or Dox-induced cell growth inhibition and induction of apoptotic cell death in both cell lines (Fig. 3D, E and F). ZnPP itself showed only a slight cytotoxicity at the concentrations used in this study (data not shown).

### HO-1 induction enhanced resistance of CCA cells to anticancer agents

**Figure 4:**
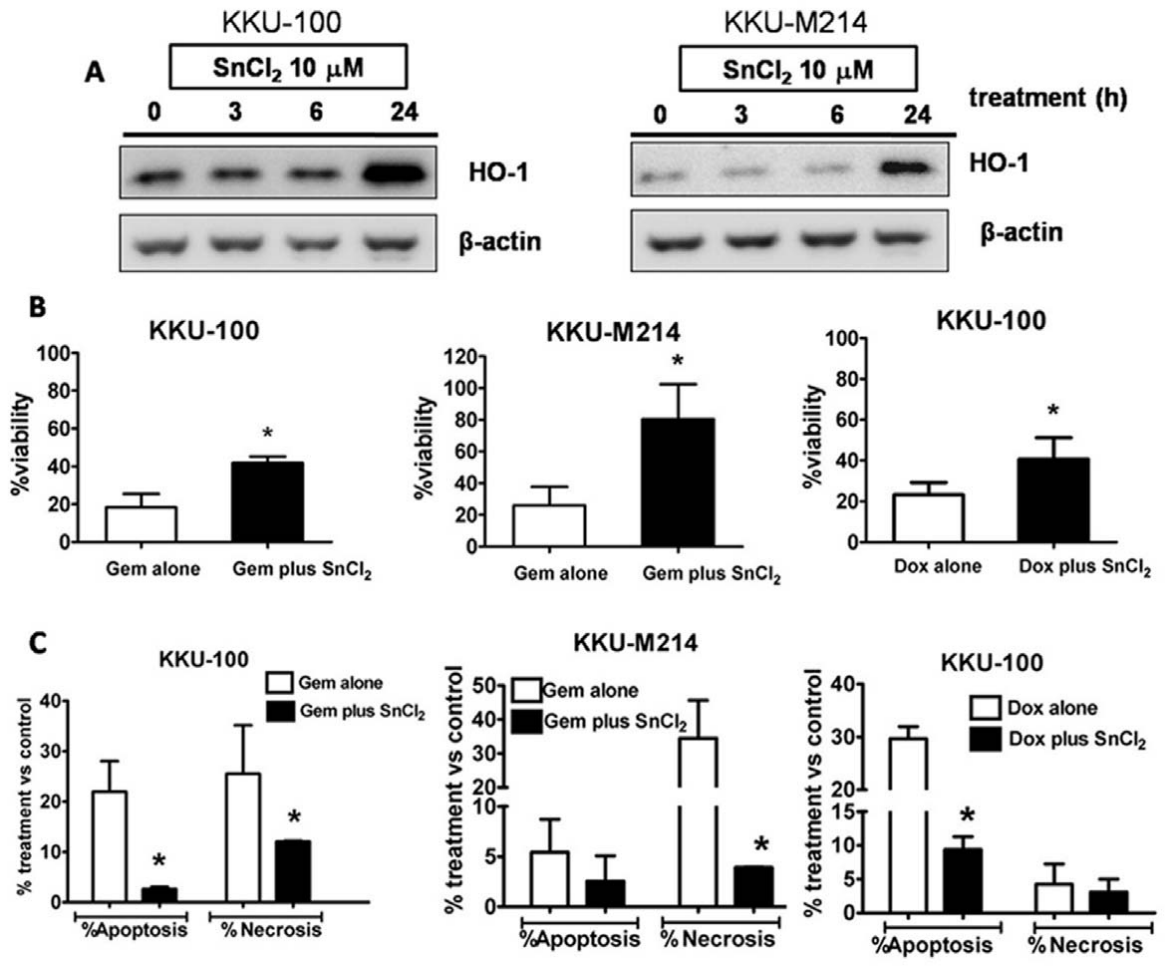
Induction of HO-1 suppressed the cytotoxicity of Gem and Dox. (**A**) The time-course of HO-1 induction by SnCl_2_ (10 µM) in KKU-100 and KKU-M214 cells. The cells were cultured for overnight and exposed to SnCl_2_ for 3, 6 and 24 h before whole cell lysates were collected and HO-1 protein was determined by the Western blotting, using β-actin; as loading control. The HO-1 protein expression in KKU-100 and KKU-M214 cells is relative stable during the first 6 h of exposure to SnCl_2_, whereas high HO-1 expression is evident during 6 to 24 h. The cytotoxicity of Gem (0.1 µM) and Dox (0.5 µM) with or without SnCl_2_ (10 µM) was determined in both cell lines after incubation for 24 h. The cell viability (**B**), apoptotic and necrotic cells (**C**) were evaluated by fluorescent dye staining. Data represent mean±SEM, each from three separated experiments. *, *p*<0.05 vs drug alone.

To validate the cytoprotective roles of HO-1, SnCl_2_, a HO-1 inducer, was used in combination with Gem or Dox. KKU-100 and KKU-M214 cells were exposed to SnCl_2_ and changes in HO-1 protein was evaluated by Western blot analysis. SnCl_2_ (10 µM) induced HO-1 protein expression with a similar pattern in both cell types with maximal induction observed during 6–24 h (Fig. 4A). KKU-100 and KKU-M214 cells were treated with Gem (0.1 µM) or Dox (0.5 µM) with or without SnCl_2_ for 24 h. Induction of HO-1 was associated with increased cell viability more than 2 fold after treatment with Gem or Dox (Fig. 4B). Induction of HO-1 by SnCl_2_ decreased the apoptotic and necrotic cell death induced by Gem and Dox in both CCA cells (Fig. 4C). SnCl_2_ alone was slightly toxic at the concentrations used in this study (data not shown).

### HO-1 gene silencing sensitized CCA cells to chemotherapeutic agents

**Figure 5:**
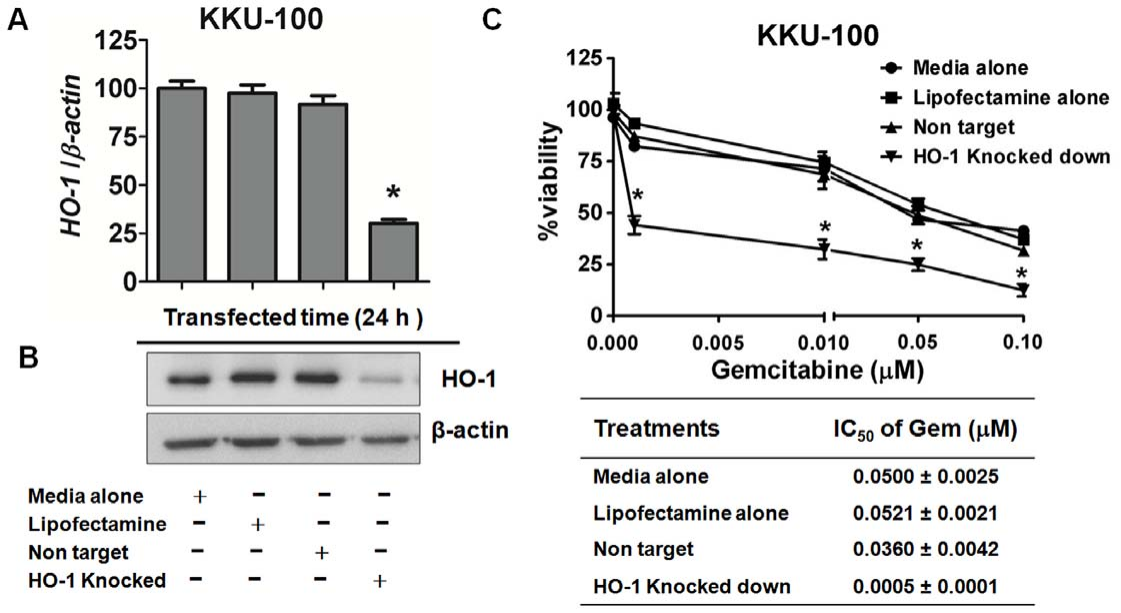
Knockdown of HO-1 by siRNA sensitized KKU-100 cells to Gem. The mRNA (A) and protein (B) levels of HO-1expression in KKU-100 cells are shown. KKU-100 cells were transfected with siRNA against HO-1 for 24 h and total RNA was prepared and analyzed by reverse transcription-PCR. In similar experiments, cell lysates were collected and HO-1 was determined by the Western blotting in KKU-100 cells, using β-actin, as loading control. (**C**) The cytotoxicity and IC50 of Gem in knocked down KKU-100 cells was determined. After transfection for 24 h, KKU-100 cells were treated with varied concentrations of Gem (0.001, 0.01, 0.05 and 0.1 µM) for another 24 h. The cell viability was evaluated by fluorescent dye staining. Data represent mean±SEM, each from three separated experiments. *, *p*<0.05 vs non target knocked down cells.

To further validate that HO-1 inhibition indeed induced sensitization of CCA cells to anticancer agents, the effects of HO-1 gene silencing to Gem was examined. Since both KKU-100 and KKU-M214 cells showed similar responses to HO-1 inhibitor and inducer, KKU-100 cells were employed as a representative of CCA cells. The levels of HO-1 mRNA (Fig. 5A) and immunoreactive HO-1 protein (Fig. 5B) were dramatically decreased 24 h after transfection of HO-1 siRNA. Then, HO-1 knocked-down KKU-100 cells were exposed to various concentrations of Gem (0.001–0.1 µM) for further 24 h. The IC_50_ concentration of Gem in non-targeting control was 0.0360±0.0042 µM, whereas that in HO-1 knockdown cells was 0.0005±0.0001 µM (Fig. 5C) (*p*<0.001), showing that the inhibition of HO-1 sensitizes KKU-100 cells to be highly susceptible to Gem.

### ZnPP induced intracellular ROS formation and mitochondrial dysfunction

**Figure 6:**
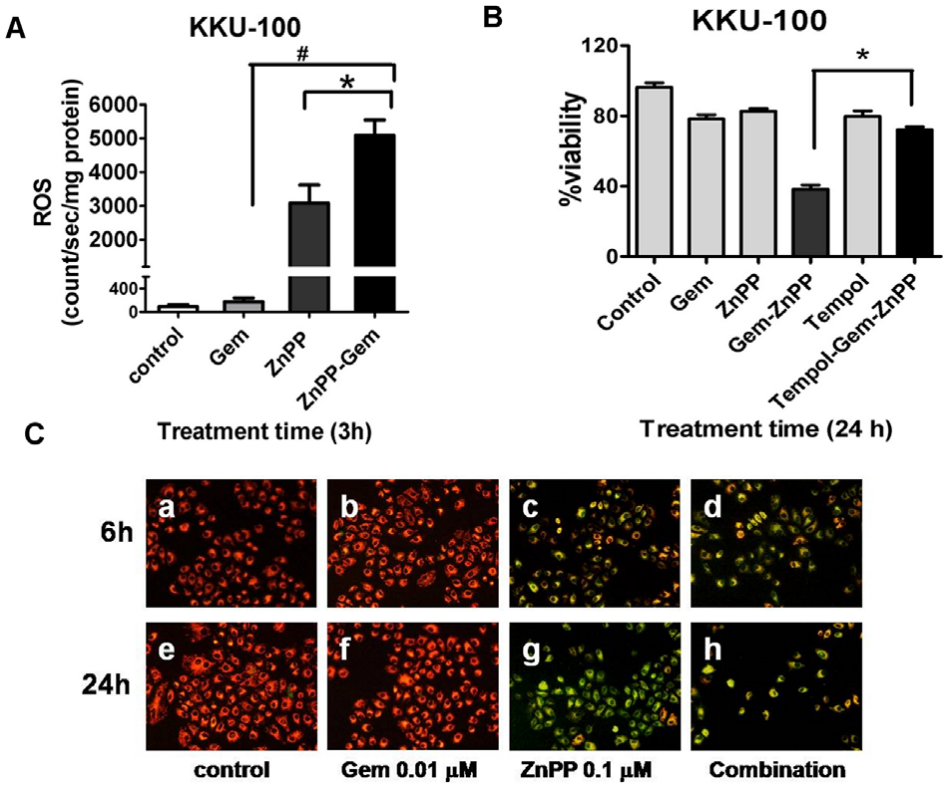
ZnPP induced the intracellular reactive oxygen generation, depolarization of mitochondrial transmembrane potential and cytotoxcity. (**A**) The generation of ROS induced by ZnPP (0.1 µM) and combination of ZnPP and Gem (0.01 µM) for 3 h in KKU-100 cells as measured by lucigenin-enhanced chemiluminescent method. *, p<0.05 vs ZnPP alone and ^#^, vs Gem alone. (**B**) The ROS scavenger, Tempol (500 µM), inhibited cytotoxicity of the combination of ZnPP (0.1 µM) and Gem (0.01 µM) in KKU-100 cells The cell viability was evaluated by fluorescent dye staining. *, *p*<0.05 vs the combination of ZnPP and Gem. Data represent mean±S.E.M., each from three independent experiments. (**C**) The induction of depolarization of the mitochondrial transmembrane potential (Δψ_m_) using JC-1 fluorescent probe, in KKU-100 cells after treatment with Gem+/−ZnPP for 6 h (a,b,c, &d) and 24 h (e,f,g & h). The healthy mitochondria, JC-1 forms J-aggregates and display strong red fluorescent signal, whereas the depolarized mitochondria, JC-1 exists as monomers and show green fluorescent signal.

From the above experiments, HO-1 inhibition by ZnPP enhanced the susceptibility of CCA cells to the cytotoxic effect of Gem. To explore the underlying mechanisms of enhanced cytotoxicity by the combination of Gem and ZnPP, the intracellular ROS formation was assessed. Treatment with ZnPP alone caused a remarkable increase in ROS formation as early as 3 h. The combination of ZnPP and Gem showed further increase in ROS levels (Fig. 6A). Gem alone showed no effect on ROS formation. To test whether the ROS was indeed responsible to enhance the cytotoxicity of Gem, the superoxide dismutase (SOD)-mimetic compound, Tempol, was used to scavenge O_2_^•−^ induced by combined ZnPP and Gem treatment. The concentrations of Tempol to be used in studies were evaluated in a previous experiment which had shown to inhibit ROS formation and produce minimal toxicity. Moreover, it is verified that Tempol (500 µM) does not inhibit HO-1 activity. ZnPP-enhanced cytotoxicity of Gem was abolished by the presence of Tempol (Fig. 6B). Tempol alone has little cytotoxicity to the cells. Since ROS formation is thought to be involved in induction of cell killing in association with mitochondrial pathway, KKU-100 cells were treated with the combination of ZnPP and Gem and the Δψ_m_ were evaluated using JC-1 assay. Gem treatment alone had no effect on the Δψ_m_ (Fig. 6 C, inset b & f), whereas ZnPP and the combination of ZnPP with Gem induced the depolarization of Δψ_m_ as evident by the change of red fluorescent staining in healthy mitochondria (Fig. 6C, inset a & e) to green fluorescent staining in depolarized mitochondria (Fig. 6C, inset c, d, g & h).

### Combination of ZnPP and Gem altered the expression of proteins related to cell proliferation and apoptosis

**Figure 7:**
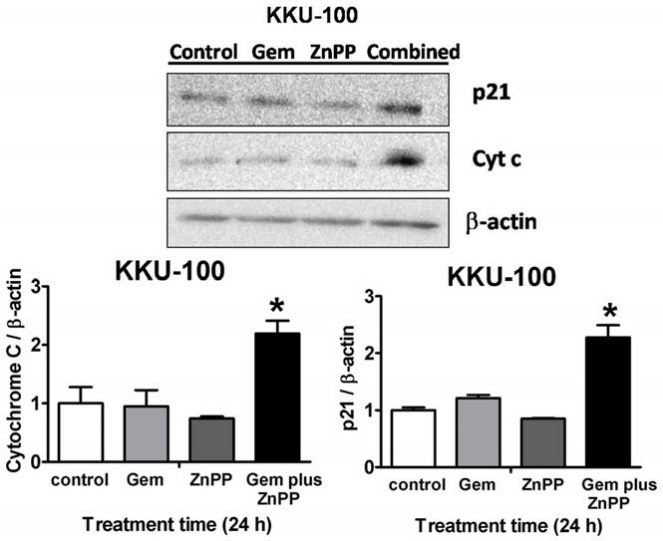
Alteration of expression of proteins related to cell proliferation and apoptosis. The Western blots of p21 and cytochrome c protein expression in KKU-100 cells after treatment with Gem (0.01 µM)+/−ZnPP (0.1 µM) for 24 h. The levels of p21 and cytochrome c were normalized with β-actin as a loading control. The bars are mean±SEM, each from three independent experiments. *p<0.05 vs untreated controls.

To investigate further the effects of combined Gem and ZnPP if it was mediated via mitochondrial pathway, cytochrome c, release from the mitochondria in response to pro-apoptotic stimuli, was determined. The combined drug treatment exerted significant increase in levels of cytochrome c protein when compared with the controls. ZnPP or Gem alone did not induce the release (Fig. 7). Since the combined Gem and ZnPP suppressed the tumor cell growth, a protein related to cell proliferation; the p21^Cip/WAF1^ was analyzed by Western blotting. Gem or ZnPP alone did not affect the p21^Cip/WAF1^ protein expression, whereas the combination of Gem and ZnPP caused marked induction of p21^Cip/WAF1^ and was associated with marked antiproliferative effect.

## Discussion

HO-1 has a potent cytoprotective activity against noxious stimuli in inflammatory diseases such as sepsis, inflammatory bowel disease, or in various oxidative injuries [7]. Moreover, HO-1 plays a protective role in normal tissues as well as in cancer cells [22], [23]. Our study demonstrated that HO-1 plays a critical role in CCA cells in cytoprotection against anticancer agents, regardless of the basal HO-1 expression levels of the cells. Inhibition of HO-1 by ZnPP or HO-1 siRNA could sensitize CCA cells to the cytotoxicity of anticancer agents, whereas the induction of HO-1 exerted a cytoprotective and drug resistant effects. The sensitization of CCA cells by HO-1 inhibition is probably involved with generation of ROS, which leads to the loss of ΔΨm, and initiates apoptotic cell death processes.

Altered expression of drug metabolizing enzymes, such as *UGT1A*, *UGT2B*, *SULT1C* [24], *NAT2* [25] and *GSTO* [26] have been reportedly associated with intrahepatic cholangiocarcinoma in endemic area of liver fluke infection. However, there is still no evidence to establish their roles in protecting cancer cells. Elevated expression of HO-1 has been reported in various human tumors including renal cell carcinoma, prostate tumors, bladder and pancreatic cancers [15], [27], [28], [29], with close association to the disease states. In the present study, HO-1 induction by SnCl_2_ caused a significant cytoprotection against chemotherapeutic agents regardless of basal HO-1 expression. On the other hand, treatment with anticancer agent also strongly up-regulated HO-1 expression, implying that adaptive defense response in CCA cells is induced to endure the drugs. These results suggest that role of HO-1 in cytoprotection is very critical even in low basal level of HO-1 expression. It should be noted that cytoprotective effect conferred by HO-1 is not specific to Gem but also to Dox and perhaps to some others, in spite of the different mechanisms of actions of these anticancer agents. These results also suggest that the resistance of CCA cells to anticancer agents is, at least in part, due to induction of HO-1. Overall, the inhibition of HO-1 may overcome the intrinsic as well as acquired resistance to chemotherapeutic agents.

Subsequently in this study, we investigated further as to how HO-1 inhibition induces the sensitization in CCA cells to anticancer agents. HO-1 possesses an indirect antioxidant effects probably via generation of biliverdin/bilirubin, carbon monoxide, and other oxidative stress responses [11], [30]. Bilirubin is a potent antioxidant by recycling between biliverdin and bilirubin during the catalytic cycle of oxidation and reduction [11]. Inhibition of HO-1 by ZnPP resulted in significant increase in ROS formation [17], [23], [29]. Our results showed that ZnPP caused marked increase in ROS levels in KKU-100 cells, whereas Gem treatment alone did not induce ROS. Moreover, combination of ZnPP and Gem enhanced more ROS production than ZnPP alone. Our study further verified that ROS is essential for the chemosensitizing effect, as scavenging of ROS by Tempol virtually abolished the sensitizing effect of ZnPP. Moreover, these results imply that inhibition of HO-1 increases the oxidative stress, where ROS may be derived from the metabolism of the cells themselves [7], [30].

The loss of Δψ_m_ is regarded as an early event of mitochondrial dysfunction. It is apparent that a small change in mitochondrial permeability transition (MPT) could depolarize the mitochondria, whilst increasing number of MPT leads to necrosis and apoptosis [31]. The increase of ROS production in ZnPP treated groups was associated with the loss of Δψ_m_. Our recent study on chemopreventive effect of curcumin has demonstrated a temporal relationship of ROS formation, depolarization of mitochondria and induction of apoptosis [32]. An inducer such as ROS may attack and modify membrane proteins of MPT pores leading to the aggregation of misfolded proteins creating regulated PT pores [31]. In this circumstance, the cells are in a state of highly susceptible to further attack by inducers of MPT. Role of ROS induced by ZnPP in relation to mitochondrial function and induction of apoptotic cell death is needed further clarification.

Alternatively, inhibition of HO-1 activity results in an increase accumulation of protoporphyrin in mitochondria [33] and this lead to depolarization of Δψ_m_ and sensitize MPT to cytotoxic agents. Protoporphyrin IX has been suggested to be a ligand of peripheral benzodiazepine receptor, a component of MPT pores, thereby sensitizes MPT [34], [35]. The present study showed that only the combination of ZnPP and Gem enhanced cell killing effect, whilst ZnPP alone at sub-micromolar concentrations caused the release of ROS in association with loss of Δψ_m_, but this was still insufficient to induce cell death. Our study is in agreement with recent reports, ZnPP alone shows no effect on MPT, however ZnPP in combination with triiodothyronine induced oxidative stress, MPT opening and apoptotic cell death [18]. Furthermore, effect of protoporphyrin IX at nanomolar range in enhanced rotenone-induced cytotoxicity is demonstrated to be due to MPT opening, because the effects were inhibited by cyclosporine A, an inhibitor of MPT [35].

The sensitizing effect is consistent with present experiment in that no increased release of cytochrome c by ZnPP or Gem alone, whereas the drug combination increased release of cytochrome c. It is noted that Gem alone may not exert cytotoxic effect via mitochondrial pathway, as Gem did not induce changes of Δψ_m_ and cytochrome c levels. Gem is known to induce cell cycle arrest and apoptosis by p53-dependent and independent pathways. [36]. Our study observed that combination of ZnPP and Gem induced an increased level of the protein p21, which is a p53-dependent downstream gene product, and a potent cyclin-dependent kinase inhibitor. Gem or ZnPP alone did not exert any significant changes in p21. It is probable that induction of mitochondrial dysfunction induces p21 accumulation [37]. This is consistent with a strong antiproliferation effect of Gem and ZnPP combination.

In summary, HO-1 plays an important role in cytoprotection in both low and high HO-1 expressing CCA cells. Inhibition of HO-1 induced ROS formation, which may initiate the loss of Δψ_m_ and sensitizes CCA cells to a cytotoxic effect of anticancer agents. Thus, targeted suppression of HO-1 may be a strategy to overcome drug resistance in cholangiocarcinoma chemotherapy.
